# New horizons in the sociology of sport

**DOI:** 10.3389/fspor.2022.1060622

**Published:** 2023-02-10

**Authors:** Richard Giulianotti, Ansgar Thiel

**Affiliations:** ^1^ Loughborough University, Loughborough, United Kingdom; ^2^ University of South-Eastern Norway(USN), Bø, Telemark, Norway; ^3^ Faculty of Economics and Social Sciences, University of Tübingen, Tübingen, Baden-Württemberg, Germany

**Keywords:** sociology, key societal challenges, sport, theory, methods, horizon, strengths and challenges of sociology, position of the sociology of sport

## Abstract

The relevance of a sociological view on the problems of society has never been as important as it is today. To quote the editors of the journal Nature in their editorial, Time for the Social Sciences, from 2015: if you want science to deliver for society, you need to support a capacity to understand that society. In other words, the technological and scientific disciplines cannot simply transfer their findings into everyday life without knowing how society works. But this realisation does not seem to have caught on everywhere. The sociology of sport is entering a critical period that will shape its development and potential transformation over the next decade. In this paper, we review key features and trends within the sociology of sport in recent times, and set out potential future challenges and ways forward for the subdiscipline. Accordingly, our discussion spans a wide range of issues concerning the sociology of sport, including theories and approaches, methods, and substantive research topics. We also discuss the potential contributions of the sociology of sport to addressing key societal challenges. To examine these issues, the paper is organized into three main parts. First, we identify three main concentric challenges, or types of peripheral status, that sociologists of sport must confront: as social scientists, as sociologists, and as sociologists of sport, respectively. Second, we consider various strengths within the positions of sociology and the sociology of sport. Third, in some detail, we set out several ways forward for the sociology of sport with respect to positioning within academe, scaling up research, embracing the glocal and cosmopolitan aspects of sociology, enhancing plurality in theory, improving transnational coordination, promoting horizontal collaborations, and building greater public engagement. The paper is underpinned by over 60 years (combined) of work within the sociology of sport, including extensive international research and teaching.

## Introduction

The sociology of sport is a relatively young sub-discipline. In the 19th and early 20th century, prominent sociologists and social psychologists, such as Karl Marx, Max Weber, Georg Simmel, Thorstein Veblen, and Norman Triplett, already discussed sport as a social phenomenon, for example with regard to the dynamics of social competition [for a detailed discussion of the history of the sociology of sport, see for example (1)].^1^ However, sport, but also the body as the instrument of competition, remained only a marginal note in sociological reflections on the changes that swept societies throughout the 20th century. One of the first large-scale works explicitly devoted to the sociology of sport was published in Germany in 1921 by the sociologist Heinz Risse. Even though the 1920s were characterized by a rapid growth of interest in sports as a topic of mass entertainment, Risse's work essentially remained an outsider's venture. The continued lack of acceptance of Risse's work in scientific circles is basically symbolic of the stereotypical devaluation of any kind of deeper scientific examination of the phenomenon of sport as a rather non-intellectual pursuit.

This marginalization of sport as an “unworthy” object of social-scientific research can ultimately be understood as the consequence of a Cartesian dualism that long anchored academic thinking. In 1641, Renè Descartes published his *Meditationes de Prima Philosophia* (English translation: *Meditations on First Philosophy*, 2008) (3) which contained the principles of Cartesian Dualism. Descartes argued that, on the one hand, physical substances (res extensae) were distinguished from mental substances (res cogitans), and, on the other hand, the body was considered only as an extended “thing” steered by volitional physical processes which are controlled by the mind. The assumption of an independence of the mind, even more, of the “I”, the subject, from a rather “machine-like” functioning body, characterized Western philosophical thinking for a long time, even among those who criticized Descartes' work. The realization that the “I” only exists as something physiological, and is therefore part of the body, was rather ignored, even though this approach was becoming increasingly prevalent in research in social-psychology and neurophysiology (3).

In the 1960s and 1970s, both the increasing sportification of society and the emerging scientification of sport, led to a growing international interest in research on sport as an important part of modern society. Numerous sociological studies, for example from Elias and Dunning (5), Edwards (6, 7), Heinilä (8), Kenyon & Loy (9), Klein & Christiansen (10), Lüschen (11, 12), McIntosh (13), and Rigauer (14), just to name a few, marked the beginning of the “take-off” of sport sociology at universities, particularly in Europe and North America, where higher education, especially in the social sciences, was experiencing significant expansion. It was no coincidence to observe during this period an accumulation of international publications on the sociology of sport from a variety of academics. Thus, in the 1960s, the discussion about the significance of sport as a sociological object of research intensified, as did the question of suitable theories and research methods for studying sport. This discussion ultimately preceded the founding of the International Committee for the Sociology of Sport (ICSS) in 1965. Clearly then, and most appropriately, the modern genesis of the sociology of sport was very much an international process, involving many academics, and carrying a strong social and collaborative impulse to advance the development of the fledgling subdiscipline.

However, even though the following two decades could be considered as a phase of establishment and consolidation of sport sociology at universities in Europe and North America, it has been a long road to gain full acceptance for sport as a subject fit for scientific study. In 1972, Eric Dunning wrote that “it is clear that the sociology of sport is not yet widely regarded by sociologists as an area posing problems of sociological importance” [(15): 101]. More than 25 years later, Dunning still saw the need to speak to this status concern, giving his sociological study of sport, violence, and civilization the umbrella title *Sport Matters* (16).

The sociology of sport shares this need to highlight and justify the importance of its subject matter with other sport science sub-disciplines in higher education, but also with physical education (PE) in school systems. Indeed, the reputation of the PE teaching profession is comparably low, sports lessons are sometimes taught by unqualified substitute teachers, while PE classes often undergo cuts in school curricula to accommodate other subjects (notwithstanding global medical concerns over the lack of physical activity among young people).

The international sociology of sport faces the further challenge that, as its subject is not only scientifically marginalized, so its scholars from different countries sometimes have differing conceptual understandings of “sport” *per se*. What is meant by “sport” is by no means unambiguous (17). In the German-speaking world, for example, even the everyday use of the term “sport” is very heterogeneous. Sport can be going to the gym, a morning jog, a yoga class, or even exercise therapy in the context of rehabilitation from coronary diseases. In contrast to the broad German meaning, “sport” is defined more clearly in the English language. Hence, for example, a more consistent distinction is made between “sport” and “physical activity” or “exercise”. The latter terms refer, often interchangeably, in common parlance to a broad spectrum of activities, such as walking and cycling through to systematic training regimes. In contrast, “sport”, on the other hand, usually refers to a form of physical activity that is characterized by an unproductive and rule-governed form of competition (cf. Caspersen, Powell & Christenson, 1985) (18). In this regard, the competitive aspect seems to be almost more significant for the understanding of the term “sport” than the physical activity, as sports such as darts, snooker and, more recently, e-sports make clear.

In line with the conceptual difference between a rather broad and a rather narrow understanding of the term, the institutional problems that the sociology of sport has to deal with are also not consistent in every respect in an international comparison. For example, networking between sociologists of sport and medical doctors, who study the benefits of physical training for heart health, may be easier in German-speaking and Scandinavian countries than in English-speaking countries, since health-oriented physical training is not necessarily an obvious subject for the sociology of sport in the Anglophone world. At the same time, we recognize too that academics may purposively seek to surmount these linguistic and disciplinary hurdles through pursuing collaborative research.

In the following, we will take a closer look at the current state of the sociology of sport, without wanting to go into too much detail about international differences. In doing so, we review key features and trends within the sociology of sport in recent times, and set out potential future challenges and ways forward for the subdiscipline.

## The challenged status of social science, sociology, and the sociology of sport: *periphery^1,2,3^*

It is not only the subdiscipline of the sociology of sport, but also the parent discipline of sociology, that continues to face a variety of major challenges with respect to its status and recognition. General concerns about the decline or demise of sociology are not particularly new: perhaps most famously, more than 50 years ago, Alvin Gouldner (18) anticipated a crisis in “Western sociology”. Yet it is our contention that these crises of sociology and sport sociology have reached particularly acute points in recent times.

It was not always so. Indeed, in the early 19th century, and prior to the founding of sociology *per se* as an academic discipline, the social philosopher Auguste Comte had envisioned that a preeminent “queen science” would be concerned with the study of human society (20). Yet, since the discipline was established, most sociologists have found themselves working in decidedly republican rather than regal times, where the prospect of ascent to an academic throne has long since been guillotined.

Here, we examine the marginal status of sociology and the sociology of sport with respect to three levels of peripherality: *periphery^1^* (as a social science), *periphery^2^* (as sociology, the discipline), and *periphery^3^* (as sociology of sport, the subdiscipline). We explore each of these levels primarily with respect to the academy, while also referring to other domains, such as policy and politics, and society and the wider public sphere.

### *Periphery^1^*: the Status of the social sciences

To begin with, in the first level of peripherality (*periphery^1^*), most social sciences have a weak status both within their universities, and in the national and international academic sectors, compared to the natural sciences. That peripherality is further weakening in several ways. On the one hand, social sciences have to compete with natural sciences for research funding. Over the last few years, there has been a tendency for social science to increasingly fall behind scientific-technological and medical projects in this area. In this context, particularly the research of newer technologies, such as AI, IoT, and quantum computing, competes with the social sciences for the distribution of funding. On the other hand, the peripherality of social sciences is manifested in its increasing replacement by the discipline of ethics when it comes to researching consequential problems of scientific-technological or medical innovations. This holds true for large-scale scientific-technological and medical research in general. Social scientific expertise is obviously not esteemed enough to become an indispensable part of corresponding projects. In contrast, there is hardly any medical research on a larger scale on societally relevant issues without the involvement of representatives from the ethics of science. The apparent omnipotence of ethical reflections is also evident in the power attributed to ethics committees with respect to the conception of research designs and thus the perspective on the phenomenon under investigation. Critics claim that the interventions of ethics committees can lead to considerable losses in quality with regard to the analytical acuity of the investigation itself [cf (21).]. Israel and Hayes even note that “social scientists are angry and frustrated. They believe their work is being constrained and distorted by regulators of ethical practice who do not necessarily understand social science research. In the United States, Canada, United Kingdom, New Zealand and Australia, researchers have argued that regulators are acting on the basis of biomedically driven arrangements that make little or no sense to social scientists” [(22), p. 1].

For medical research, the ethics of science has become a multi-purpose weapon for analyzing non-medical issues, both as part of the research group itself and also as an institution of meta-reflection on research. Thereby, it obviously does not matter that the competence of ethics of science rather lies in initiating (quite necessary) debates about relevant moral questions and providing guidance for concrete action (applied ethics) than in the systematic reflection of consequential societal problems of medical research. There is a fundamental difference between ethics and social science with regard to how scientific problems are approached. Zussman (23) argues, for example, that sociologists cannot answer normative questions that constitute the core of medical ethics, but they can provide a “realist” critique of medical ethics in practice, for example, by analyzing the reasons why physicians persistently deflect challenges to their authority or under what circumstances patients are able to autonomously decide on therapeutic options. In this sense, we do not argue for the abolition of ethical reflections on scientific, technological and medical research, but note that ethics is far from being able to cover all the questions that arise in connection with such research.

Some prominent natural scientists have obviously already recognized this when doubting that the technological and scientific disciplines can transfer their findings into everyday life without knowing how society works. A Nature (24) editorial titled “Time for the social sciences” emphasized the relevance of social scientific expertise for natural scientific and technological research. The editors stated that “governments that want the natural sciences to deliver more for society need to show greater commitment towards the social sciences and humanities” [(24), 7,532]. Summarizing the key message of the UK Government Chief Scientific Adviser's annual report for 2014 (Walport & Beddington, 2014) (25) they added that “if you want science to deliver for society, you need to support a capacity to understand that society” [(24), 7,532].

From this, we might ask: How can social science manage to make itself heard? And what type of social scientific research is best positioned to be heard? The societal environment of social sciences certainly seems to have specific expectations of their services. Both medicine and scientific-technological researchers, but also the media, which report on scientific results and their practical applicability, obviously tend to prefer relatively quantitative, causal, and predictive research findings, that are rooted in large-scale datasets, and which can, for example, provide politicians and other key decision-makers with “hard data” about prospective returns on their investments. Conversely, much of social scientific research generates qualitative, interpretive, and highly contextual findings that are usually rooted in relatively small-scale empirical studies, and which are less focused on generating predictions or policy recommendations. The challenge for social scientists, then, is to find ways of responding to these circumstances, to find explanatory techniques for engaging these audiences, or to endure continuing, perhaps even intensified, peripherality vis-à-vis the natural sciences, with all the attendant institutional consequences.

### *Periphery*^2^: the Status of sociology

A second level of peripherality—the periphery-squared or *periphery^2^*—involves the relatively weak standing of the discipline of sociology within the social sciences on the one hand and politics and policy on the other hand. In a similar way as compared to the social/natural science power imbalance, the lower status of sociology compared to a host of other social sciences such as economics, political science, and social psychology, is reflected in interrelated areas such as research funding and impact, student recruitment, the professional or career pathways that are afforded to sociology graduates, and the lack of influence of sociological research in the private and public sectors. A relative exception lies with demographers and other quantitative sociologists, whose “scientism”, in Gouldner's phrase, in regard to methods, findings, and recommendations, mirrors those within the natural sciences in ways that tend to be favoured by external research partners. Arguably in the UK and other nations, sociology has also been one of the disciplines most adversely affected by financial squeezes on social science, and on higher education more generally, which have occurred since the 1990s.

Sociology has been adversely affected by the long-standing hegemony of neoliberal social and economic policies, which emphasize individualism and self-responsibility, in marked contrast to the themes of society and social interdependencies that underpin much sociological scholarship. Additionally, there are few if any sociologists who can justifiably be described as public intellectuals in terms of social profile and influence. Arguably the situation has worsened since the 1980s and 1990s when Ulrick Beck, Pierre Bourdieu, Anthony Giddens, and Jurgen Habermas exercised significant presence in political and wider public debates. The lack of awareness of sociologists and sociological research by policymakers may also stem from sociology's failure to generate public interest. The observation that “Sociology is only marginally recognized by its own subject: society” describes this problem very accurately. Sociology generates a lot less social and political resonance than it actually should. This became abundantly clear during the Covid-19 pandemic, when its causes and consequences were almost entirely considered from a medical-scientific perspective, more precisely by virologists and epidemiologists. In contrast, the social consequences of the pandemic were as much neglected as its social dynamics. Certainly, questions with social scientific relevance were raised by both health policymakers and journalists. For example, there were strong discussions on how to allocate intensive care beds in the event of insufficient capacity, taking into account socio-economic and educational inequalities. Another topic concerned socially just vaccination priorities, considering the assurance of medical care, the issue of maintaining the economy and work vis-à-vis pandemic lockdowns, and the provision of cultural and leisure activities. Not least, critical journalists asked how medicine can meet the needs of socio-economically disadvantaged groups in the pandemic, or to what extent high-income countries should support low-and middle-income countries in coping with Covid-19 and its consequences. All these questions have direct thematic relevance to the core area of sociology. However, despite some exceptions, sociology has obviously not succeeded in convincing politicians or medical, epidemiological, and virological scientists of its particularly well-developed theoretical and methodological competence for analyzing the most complex, interconnected, and societal problems.

Two further points might be made here on the factors that lurk behind sociology's limited purchase in policy and public domains. First, the self-referentiality of sociology may be one hurdle. The prominent sociologist Peter Berger once said that “it is fair to say that the first stage of wisdom in sociology is that things are not what they seem” (2011: 41) (26). Sociological theorizing does not have a practical value *per se*. To critically reflect on everyday theories and to “de-construct” popular interpretations of patterns within social phenomena is a merit in itself. However, critical reflections produce little effect if they do not reach the public. In sociology itself, however, the question of how to generate political and/or public interest, seems to be discussed rather little. Rather, discussions on science-policy are largely limited to (often self-defeating) arguments about methodological paradigms (e.g., qualitative vs. quantitative research), the appropriate degree of advocacy (e.g., critical vs. descriptive-explanatory research), or basic epistemological questions (e.g., anti-positivism vs. positivism). The continuous questioning of competing theoretical models for the description and explanation of social phenomena and empirical methods for their recording is certainly necessary to keep pushing the discipline forward. From a social scientific perspective, this makes sense because critical thinking is an essential prerequisite of systematically “scrutinizing” theoretical assumptions, and replacing them with theories that carry a higher explanatory power. Positively speaking, sociologists cultivate “a kind of “art of distrust” (not only) towards the self-evident facts of everyday life” (Eickelpasch, 1999: 10) (27), but also towards the fruits of their own creations. More problematically, for non-sociologists, these important practices may resemble a form of obscure sociological navel-gazing that has no obvious beneficial outcome.

Second, and in part following from this, sociologists may also appear to be unduly preoccupied in some contexts—especially in German-speaking countries—with often fractious and inconclusive debates on the status or meaning of “critical thinking” within their discipline. The discussion on the extent to which sociology may engage in “advocacy” goes back to Max Weber and received special attention through the controversy between the sociologists Jürgen Habermas and Niklas Luhmann. This dispute was basically about whether it is sufficient for sociology to limit itself to describing how society changes, but not how it should change. (Note: here, in line with the philosophical tradition of “critical theory”, the term “critical” refers at least in part to the advocacy of social change and to envisioning alternative ways in which society should be organized.) The criticism of an “apolitical” sociology was that an exclusively “uncritical” sociology could not initiate any necessary social changes but would ultimately have a rule-legitimizing function. Luhmann's counterargument was that (normative) criticism of existing conditions leads to hasty judgments. Thus, the attempt to prove the possibility of a “better” society fails because of the complexity of the world; accordingly, criticism falls into inconsequential humanity. We return to this question of critical thinking later on, but here, the key point is that, to outside observers, sociologists fail to communicate the significance of such debates, and thus appear overly distracted with such concerns. In this sense, sociology is confronted with the dilemma of the simultaneous need for analytical value freedom and inspiration for social change. On the one hand, there are political, policy, public, and, in some areas, philosophical expectations that the “critical” standpoints of sociologists should include normative sketches of alternative social arrangements. On the other hand, however, there is the counter-expectation that such normative statements automatically fail to encapsulate or to account for the complexity of society. This latter position further contends that, to the extent that sociology claims the competence to make normative statements, it inevitably disavows its scientific analyses. From these types of debates, we would highlight the broader point, that the “critical” is understood in diverse ways within sociology, and that such diversity is indicative of the vitality of the discipline, and also its positive capacity to investigate and to engage with social phenomena in a variety of ways.

### *Periphery^3^*: the Status of the sociology of sport

All of these challenges are magnified when we move from the positions of social science, and of sociology, to examine the specific standing of the sociology of sport, which occupies a third level of peripherality—the periphery cubed or *periphery^3^*—within academe, as well as in other, non-academic domains.

In academe, there are dual challenges for the sociology of sport, in its overlapping positioning within the fields of sociology and sport studies. On one hand, within the general sociological community, the subdiscipline's struggle for recognition and credibility is evidenced by the rarity with which it variously is taught or researched within mainstream sociology departments; contributes papers to leading sociology journals, particularly in the United States; and secures significant levels of competitive research funding from major foundations. At the same time, the topic of “sport” in general sociology tends to be a pastime for scholars who otherwise deal with topics such as social inequality, the evolution of the financial system, the family, or conflict, and so on. To adapt Rowe's (25) observation of sports journalism within the news media, sociologists have long tended to view sport as the “toy department” of their discipline, in marked contrast to deeply established subject areas, some of which, such as religion, have been in long-term decline in many late modern societies. This corresponds with the fact that chairs designated for sport sociology are at many universities either nonexistent or still located in institutes of sports science. Hence, one could say that the institutional problems with which the sociology of sport must deal have changed less than we representatives of the subdiscipline might wish.

On the other hand, in sport studies, the sociology of sport faces a further set of challenges at two main levels. First, at the level of *periphery^1^*, sociology and the other social sciences tend to have relatively marginal statuses in sport studies overall. For example, the natural rather social sciences tend to hold greater influence and presence in many departments or schools that focus on sport, physical activity, and/or exercise (or “kinesiology”, in North America). They are also viewed—by schools, faculties, and universities—as much better placed than the social sciences for attracting students, research, and enterprise income, and for influencing policy and practice within the sport sphere. Second, at the level of *periphery^2^*, within the social sciences of sport, sociology also faces significant challenges. Other social sciences in sport—such as sport management and those in the business spheres—are seen as having greater practical and vocational relevance, and are able to attract more students, particularly international postgraduates, by offering more direct entry to preferred employment and careers. These developments reflect a wider criticism that the sociology of sport has been slow to respond to the large and rapid expansion of the global “sport industry” since the 1980s.

These challenges have long-term consequences for the sociology of sport within academe. They threaten the volume and quality of funded research, and subsequent publications, within the subdiscipline. Many students (as future academics)—whether on sociology, social science, or sport studies undergraduate or postgraduate degree programmes—have relatively fewer opportunities to study the sociology of sport in some depth and detail. Hence, we find that many of those whom we do attract into the sociology of sport—such as PhD students, association members, and prospective contributors to subdiscipline journals—have not had the benefit of an initial, substantial grounding in the subdiscipline or in the parent discipline of sociology.

In turn, the sociology of sport finds itself in a recruitment dilemma. On the one hand, young sport sociologists need to complete their qualified training in sociology, to know and be able to apply the most important theories and methodological approaches on sport specific phenomena. On the other hand, sport is a highly complex subject that cannot be adequately understood by only observing sporting events, as some sociologists and economists still claim today. To analyze sports in a competent scientific manner, sports sociologists also need at least a basic understanding of wider sport-related issues and processes, such as how movement and training processes work, how tactical systems evolve, or what the motives of different population groups are for doing sports. Hence, an education in sports science makes perfect sense. Yet, alone, it is not sufficient for research in the sociology of sport. If young researchers in sports sociology are recruited from sports science, kinesiology, or physical education, then they must therefore acquire sociological knowledge during their doctoral studies, just as sociologists without sports science training would benefit from familiarizing themselves with other disciplines within the sport and physical activity fields, such as exercise physiology, biomechanics, sport psychology, and sport pedagogy.

Overall then, the sociology of sport finds itself in a position where three layers of peripherality (as social science, discipline, and subdiscipline) are in play. In passing, we might note too that these insights provide an uncomfortable contextualization to any references to “stars” within the subdiscipline. As sociologists, we consider it important to set out the context in which the subdiscipline is located before turning to discuss the strengths and potential ways forward for sociologists of sport.

## Strengths in the position of sociology and the sociology of sport

We may highlight some of the potential strengths and positive aspects of sociology and the subdiscipline of the sociology of sport vis-a-vis academe and in wider non-academic contexts.

First, the fundamental premise of sociology should be viewed as a core strength in securing and enhancing the discipline's academic and wider standing. In 1987, the UK Prime Minister, Margaret Thatcher, opined that, “there's no such thing as society. There are individual men and women and there are families.”^2^ In contradistinction to this New Right, neoliberal credo, sociology is the academic discipline that, more than any other, reminds us that there is such a thing as human society. There are very strong audiences for that social philosophy in most if not all societies. Moreover, it is also a central tenet of most social sciences.

Second, as we have indicated earlier, the diversity of critical dimensions of sociology, and the sociology of sport, represent a further positive. The task of sociology is not to substantiate what seems to be self-evident, but to reveal the contradictions inherent in it. In this sense, the rejection of critical analysis of social reality, with reference to Weber's postulate of value freedom (26), is based on a misunderstanding. Critical thinking also has a function from a Weberian perspective, for example, to the evaluation of a means to fulfill a purpose, i.e., whether the use of a means is appropriate to that purpose. To think “critically”, however, from this perspective, should not mean to base sociological analysis on premises foreign to science, for example, on politically motivated *a priori* distinctions of “good” and “bad”. In this sense, by critical, we we are referring to what sociologists sociologists, in the course of their analysis of academic literature and while undertaking social research, should focus on: de-constructing any errors, misunderstandings, inconsistencies, and contradictions that may be identified in the scientific, politic, medial, and public descriptions of social issues; examining the key features and patterns of social relations; comparing and contrasting, and identifying strengths and limitations, in theories, policies, and patterns of social relations; highlighting and investigating social relations of power, as characterized for example by social inequalities and divisions; and, identifying alternative possibilities for how societies may be organized, including within particular areas of social life, such as in sport. This type of critical ethos within the discipline has strong resonance across diverse social groups, who are both curious and furious about how sport and wider aspects of society are organized, and how power is unequally distributed in ways that lead to marginalizing and depriving outcomes for many.

Third, we appreciate also that sociology has consistently been an avant-garde discipline, in terms of identifying and highlighting progressive public issues that go on to gain some traction with wider publics, policy-makers, and corporations. Areas such as EDI (equality, diversity and inclusion) and ESG (environment, social and governance)—that are rooted in themes relating to social division and social justice, which have long been a major concern for sociologists—are illustrative of this avant-garde impulse. Sociologists had been highlighting forms of racism, sexism, homophobia, and other forms of social abuse, discrimination, and intolerance within sport long before these were addressed as serious social issues by most sport authorities. There is then the need for sociologists to continue exploring progressive new domains of research and social commentary, where they may have future influence. One approach here would be for (sport) sociologists to consider alternative possibilities for the social organization of sport for two decades' time, and to think about what social roadmap would be required to get there.

Fourth, the plural, diverse, and in many ways diffuse disciplinary nature of sociology is a strength. Unlike some other subjects such as economics or law, which rather restrict entry into their respective academic fields, sociology has been and continues to be open to diverse disciplinary contributions and influences. This is very much a two-way street: sociology has always bled into, and been significantly shaped by, other disciplines, particularly related ones such as anthropology, education, history, human geography, political science, social policy, and social psychology. Sociology is also a core constituent of many of the transdisciplinary “studies” domains, such as the vast field of cultural studies, which to a large extent encompasses other, more specific fields such as gender studies, race and ethnicity studies, and LGBTQ+ studies; as well as in the similarly vast, if rather different domain of “business studies” or “management studies”. Particularly in management studies, there is reason enough to apply sociological knowledge when analyzing the organization of sport. Many sports organizations, for example, are not commercial enterprises but voluntary organizations. However, blindly applying economic concepts to volunteer organizations negates the fact that the two types of organizations follow completely different operational logics (27). On the other hand, intellectual exchanges and collaborations with these other disciplines and transdisciplines help to invigorate and to revitalize sociology, through the infusion of fresh research theories, methods, and paradigms. They also highlight how sociology's influence in academe may be relatively broad and diffuse, reaching well beyond the formal (and, usually, shrinking) realms of academic departments of sociology.

Fifth, following from this, we may identify a diffuse influence of sociology within wider non-academic spheres—in politics, social administration, media, business, civil society, and so on. The point here might be more clearly made if we differentiate between “capital S” Sociology, representing the institutionalized master discipline as practiced by recognized, professional sociologists, often operating within named Sociology departments; and “small s” sociology, as practiced by anyone who draws upon sociological ideas, keywords, principles or themes, even without recognizing their formal association with the discipline of sociology *per se*. This connects to the earlier points on the avant-garde aspects of sociology, in fields such as social inclusion. It is here, in “small s” sociology, that the discipline might exercise its best influence, such as through feeding sociological themes and approaches into diverse degree programmes, research projects, policy analysis and guidance, and public debates.

Sixth, the sociology of sport has a particular need to be open to transdisciplinary views on the phenomena it is dealing with. Due to the complexity of the subject of sport and due to the necessity of frequently also having to consider economic, psychological or even physiological aspects when analyzing the sport of society, sociologists of sport have to be generalists in a certain sense. The advantages of the generalist perspective are at least two-fold. On the one hand, it ensures that the problems of sport, which are usually very complex and demand multidisciplinary study, can be understood as a whole. On the other hand, researchers in the sociology of sport are also predestined to look beyond the confines of their own subdiscipline, which in turn makes it easier to collaborate with colleagues from other scientific disciplines.

Seventh, sociologists of sport have to find ways to secure positions within academe. These prospects continue to be squeezed by the contraction and in some cases closure of sociology departments, research units, and degree programmes for a variety of stated reasons. In response, many sociology units have innovated by connecting or combining with other disciplines—such as criminology or social policy—which appear to attract more students and/or research funding.^3^ In sport studies, the most obvious partner discipline is sport management, which tends to attract larger cohorts of students, particularly at postgraduate level, while affording opportunities for collaborative research and teaching, notably in areas such as social inclusion and sport for development. Indeed, it may be that such a necessary, pragmatic approach will involve “small s” rather than “capital S” sociology continuing to operate in sport studies degree programmes or departments. For example, while named “Sociology of Sport” degree programmes may be closed due to low student recruitment, it may remain feasible to feed sociological content into courses at more everyday levels through lectures and seminars. Such innovative responses will vary by context—particularly along national or regional lines, where the discipline and subdiscipline will encounter different pressures and potential opportunities—but are likely to continue to be required at least in the medium term.

## Ways forward for the sociology of sport

We have discussed in detail the problematic status and other challenges that face sociology and the sociology of sport, as well as various strengths in their positions particularly within academe. It is appropriate now for us to turn here to consider some of the ways forward for the discipline and subdiscipline in this regard. There are several ways in which sport sociologists may respond here, and we begin by assessing their positionings within academe.

### Positioning within academe

First, the theme of interdisciplinarity in academic work has been advocated, celebrated, and even fetishized for several decades; it has also been heavily commodified through the allocation of funding—from small travel grants through to multi-million Euro research programmes—to those who commit to undertake such work. Moreover, universities are increasingly set up to facilitate such work, notably through interdisciplinary research centres and Institutes for Advanced Studies. Here, we echo these calls for interdisciplinary activity, but would add that such work involving sociologists needs to be adventurous and open-ended wherever possible, involving for example looking beyond close, cognate disciplines (such as anthropology, history, political science) to explore collaborations with a wider array of disciplines, including in the natural sciences. The structure of sport studies departments—in which the social and natural sciences coexist—provides comparatively favourable ground for exploring such collaborations. One potential consequence is to enable sociologists to be more actively engaged in high prestige, large scale, and heavily-funded research programmes that tend otherwise to be fully dominated by the natural sciences.

Second, to build on our points earlier, we note the need for the sociology of sport to engage with other academic disciplines and subdisciplines in open, collegiate, mutually beneficial ways. On one hand, there is the concern to enhance the full participation of sociologists of sport within interdisciplinary, multidisciplinary, and transdisciplinary research projects and other academic initiatives. Such collaborations across disciplines have come to dominate the research funding landscape, hence the subdiscipline needs to follow this path for strategic as well as for intellectual and wider academic reasons. On the other hand, sociologists of sport would do well to engage more with, and to gain enhanced inspiration from, the broader, parental discipline of sociology. This would enable the subdiscipline to draw more fully on emergent and diverse sociological theories and methods; to highlight the work of prominent “mainstream” sociologists (such as Wacquant) who engage with sport; and, to draw more of these scholars into projects and papers on the sociology of sport. These wider engagements would serve to underline the legitimacy, significance, and vibrancy of the subdiscipline, and to start to tackle its peripherality, vis-a-vis wider communities of scholars in sociology and social science.

Third, sociologists, whether in sport or in other fields, would do well to maximize their social, cultural, and political capital within academe. University leadership roles—such as Rectors (the head of universities), Deans (of Faculties), and Heads of School—provide important positions that, *ceteris paribus*, may serve to safeguard the interests of sociology and other social sciences, when alternative leaders, drawn from other disciplines, may be decidedly more skeptical or even hostile. Further beneficial leadership roles in this regard include those within national and international academic associations and networks, particularly those that encompass a wide spectrum of social sciences or both social and natural sciences; and those that offer formal connections between the academy and important external organizations, such as with global sport governing bodies or UN agencies.

Fourth, in part to enhance its positioning within academe, the sociology of sport needs to be agile, inventive, and relevant in both the research that it undertakes, and in its external activities. Sociologists benefit from commitments to investigating fresh substantive areas, particularly given that sport is constantly being shaped and reshaped in economic, social, cultural, political, environmental, and technological terms. Such a research approach is more likely to enable sociologists of sport to collaborate with other disciplines that are concerned (and, often, funded) to investigate cutting-edge issues. The development of original research is also significantly enhanced if sociologists of sport engage with and potentially draw upon innovative aspects, in theory and in substantive research, within the parent discipline of sociology as well as in other disciplines or fields, such as anthropology, cultural studies, development studies, geography, international relations, and political science. Further benefits can only accrue from continuous self-critical inquiry, asking for example, what fresh theories, methods, concepts, keywords, research topics, and pedagogical techniques might be explored by us. The alternative approach—involving an instinctive, even institutionalized reluctance to explore fresh thinking—not only makes for a stultifying and boring subdiscipline. It also makes the subdiscipline appear somewhat ossified to our colleagues in mainstream sociology and other disciplines—and thus, far less likely to be considered as a worthwhile research collaborator.

Fifth, all research fields, as international communities of practice, prosper when diverse scholars engage in collegiate collaborations, and in open and temperate debates. The sociology of sport has many such examples involving teams of scholars who operate within and/or across different institutions, for example in teaching units and research projects, or in collaborative publications and gatherings at conferences. As new generations of scholars emerge, often without lifelong commitments to “defending” fixed theories and paradigms, there also appear to be fewer vituperative exchanges or interrelations than in the past few decades. Moreover, in the post-Covid academic environment, we detect strong atmospheres of friendly sociality and restored community within at least some sociology of sport gatherings. It is vital that the sociology of sport builds on such collaborative and collegiate activity to safeguard the subdiscipline.

### Scale up: towards large-scale research collaborations

The sociology of sport, and indeed the wider social scientific study of sport, continues to do research that is mostly qualitative and relatively small-scale, and which commonly features individual studies of specific groups, communities, or organizations with reference to involvements in sport or physical activity. Much of this work also reflects a “methodological nationalism”, in terms of empirical focus, research team collaboration, and/or academic reference points. Even comparative studies continue to be small scale, usually focusing on a handful of research groups or locations, while engaging relatively small research teams. This stands in marked contrast to much quantitative research, especially in the natural sciences, which has the capacity to generate much wider-reaching data, and benefits increasingly from technological advances that allow for rapid large-scale data production and processing, and for the meshing of multiple datasets. Such research is also more likely to be undertaken or written up by relatively large teams of researchers, who may each contribute their own data sets, or diverse types of expertise for producing and analysing data—hence, the large numbers of co-authors that we find on many quantitative papers. Furthermore, this large-scale approach carries appeal for many grant-making foundations and external stakeholders—whether in policy, commercial, or civil spheres—in terms of promising findings with relatively greater reach, reliability, and validity, which may in turn guide investments and other strategic actions by key decision-makers.

Here, we call for academics and students in the sociology of sport, particularly those working with qualitative methods, to consider how they may “scale up” their research activities and aspirations. By “scaling up”, we are referring to various potential actions, most obviously the extensive enlargement of research teams, and/or a substantial increase in the number or variety of social groups or locations that are the focus for research. There is, then, every reason for scaled-up research in the sociology of sport to engage research teams of 20+ scholars working in a similar number of locations. Such scaling up of research teams and research designs would enable sociologists of sport to undertake challenging programmes of research that would aim to generate findings that are richer in content and depth, more rigorous in how they have been produced, more comprehensive in their reach and scope, and more influential for future researchers and external stakeholders. This would, for example, enable sociologists of sport to respond more effectively to calls by officials within government and civil society for research findings that are sufficiently specific, detailed, and wide-reaching, and which provide the basis for guiding key decision-makers on how to construct policy and on how to invest money and other resources in different areas of sport.

We may observe too that scaling up would enable sociologists of sport to contribute more fully to enlarged, interdisciplinary research programmes. A problem that has received little attention to date, but is all the more relevant and can only be adequately addressed by larger interdisciplinary teams, concerns the mechanisms of interaction between the social and the biological. In terms of research methodology, there are as yet only few multidisciplinary explanatory models of how the diverse, elusive, and chaotic, and thus ultimately unpredictable, environmental influences interact with biological adaptations at the epigenetic level (31). However, there is certainly reason to believe that social structures and social regulations are directly and causally linked to genome structures and gene regulation (32). For example, studies indicate that nutrition in early childhood, on the one hand, conditions metabolic structures at the molecular level, which in turn have an effect on nutritional physiology in adulthood (33). On the other hand, nutrition in early childhood is in turn, simply put, dependent on the parents' attitudes toward nutrition, the extent to which they have the educational prerequisites to distinguish between healthy and unhealthy food, what food is available in the first place, and what food the parents can afford in light of their economic situation. It can also be assumed with regard to individual sports activities that being socialized into sedentary living conditions leaves traces not only on the attitudinal level of people, but also in their biological makeup. Within scaled-up and interdisciplinary research programmes, the sociology of sport, together with sports medicine and epigenetics, could well contribute to finding explanations of how the “sportive body” develops in its unique, ever-changing relationships with the world, and how biological systems react to environmental influences and in this sense “learn” in a rudimentary way (31, 34).

The sociology of sport has the professional, social, and technological infrastructure to scale up its research. Many of the research fields within the sociology of sport have a substantial critical mass of scholars located across the world. Each of these scholars will have their own networks of research groups that they study, and fellow academics with whom they tend to collaborate. A scaled-up set of research collaborations would be facilitated by a “network of networks”, drawing together these different groupings. We also have the online technologies and experience for making research collaborations viable online. The routine use of online communication platforms (Zoom, MS Teams, Google Meet et al.) during the Covid-19 pandemic demonstrated how social science research and teaching, engaging large numbers of participants, could be successfully undertaken through virtual technologies. The return to normal academic life—albeit, still, an uneven and incomplete process—has been a positive, social experience for many, marked for example by strong senses of community such as at international conferences and other gatherings.^4^ Arguably, then, the post-Covid camaraderie within the academic community, which we noted earlier, provides relatively auspicious ground for the scaling up of research. Finally, a host of core themes in contemporary social science—relating, for example, to globalization, development, postcolonialism, decolonization, and EDI (equality, diversity, and inclusion)—has pressed the transnational academic community, still dominated by global North, to explore ways in which academics, students, and institutions in the global South may become full leaders and participants within world academe. The process of scaling up will require sociologists of sport to ensure that the global South is much better engaged in shaping research issues and designs, and in contributing to and leading research teams.

We may pick one research field, by way of illustration. Sport for development and peace (SDP) has mushroomed into one of the largest, genuinely global research fields in the sociology of sport and related subdisciplines over the past two decades.^5^ Yet, most academic work in SDP continues to involve qualitative research that is relatively small-scale, both in empirical focus and reach, and in the composition of research teams. To scale up, the field of SDP research may establish a large transnational team of academics—why not 20–30 scholars?—drawn from the global South and North, pulling together their diverse research networks, to undertake a systematic programme of research across the world, focused on a common set of research issues and questions. This scaled-up research would be best placed to drive a step-change in SDP studies, providing research findings with new levels of reach and significance than hitherto, and offering a potential model for research programmes in other fields of the sociology of sport.

With regard to collaborations with researchers from other disciplines, one has to keep in mind that it is not a matter of course that the participants of an interdisciplinary research group are able to understand the language, methodology, and operational logic of representatives of other disciplines. Disciplines are *per se* autonomous and operationally closed systems that cannot simply exchange knowledge without translation work [cf (36).]. Cross-disciplinary collaboration requires an understanding of the theories, methods, and practices of dealing with knowledge gained in each other's disciplines, but also an acceptance of the scientific value of the knowledge produced in the “foreign” discipline. Hence, researchers from different disciplines involved in an interdisciplinary knowledge production process do not necessarily recognize or understand the object under analysis in the same fundamental ways. Thus, in any inter- and transdisciplinary work, attention also needs to be given to the “translation” that occurs between disciplines. If this translation work is not part of the process of knowledge production, then any forms of “interdisciplinary cooperation” will, in reality, be restricted to adding single disciplinary findings to an additive “multidisciplinary” bundle.

### Embrace the glocal and cosmopolitan aspects of sociology

We appreciate that the sociology of sport, like the overarching discipline of sociology, has a largely glocalized academic status. In other words, while sociology and the sociology of sport constitute a global discipline and subdiscipline respectively, their shapes and statuses can vary significantly by national or regional context.^6^ In much of Europe and North America, as we have outlined, the sociology of sport has been heavily marginalized by neoliberal policies, the marketization of higher education, and late modern ideologies and cultures of acquisitive individualism. The stronger presence of the public sector in higher education in some contexts, notably in France or Germany, can work to protect sociology's role to some degree. Significant cultural differences also arise. In the United States, quantitative sociology has greatest traction. In France, sociologists contribute prominently to social and political debates in the public sphere. In Latin America, social sciences, including in the sociology of sport, have tended to convey relatively direct and extensive forms of oppositional political critique—reflecting decades of structural crises, and academic activism against authoritarianism and social injustices—alongside adventurous and expansive forms of social and historical analysis. In other regions—such as in East Asia—the sociology of sport tends to be relatively well represented within sport-focused departments and universities, in part reflecting institutional commitments to housing a comprehensive array of disciplines.

The glocal aspects of sociology and the sociology of sport—particularly in how the discipline and subdiscipline are understood and performed with respect to theory and method—should be strongly embraced and nurtured. Such glocal processes reflect how sociologists, with diverse cultural and other backgrounds, seek to apply and develop the discipline and subdiscipline, in ways that are most meaningful and applicable within their different locations and traditions of scholarship. They protect and sustain the cosmopolitanism of sociology, and of the sociology of sport, by recognizing and valuing cultural “difference”, in this case with regard to the plurality of sociological perspectives *per se*. Further, these glocal and cosmopolitan aspects are in line with calls for global sociology to advance the voices of relatively marginalized approaches and perspectives, such as those from low- and middle-income countries (LMICs) and from non-Anglophone cultures (see our further comments, below). Ideally, they should also enhance greatly the vitality of the discipline and subdiscipline, by enabling diverse approaches and perspectives to commingle—such as through research projects, publications, and conference debates—in ways that inspire further, original work, in theory, method, and empirical inquiry.

### Theory: plurality, and fresh approaches

Following from this, we contend that it is important for any discipline or subdiscipline in the social sciences to have as wide a range of theoretical and methodological techniques at its disposal as possible, so that in social research the most appropriate theories and methods may be used, to the greatest effect, in order to study, analyse, and explain social phenomena or processes that are under investigation. In addition, theoretical and methodological diversity and innovation represent important indices of the health and vitality of any social science. Fresh theoretical developments point to a vibrant academic community, whereas little conceptual innovation suggests a discipline that is staid if not entropic.

The sociology of sport has an uneven position in regard to theory. On one side, the subdiscipline has a long history of diverse theoretical approaches that have been utilized, often with significant variations by nation or region. Further theoretical range is afforded by referring back to the master discipline of sociology, and by engaging with cognate disciplines that often have significant sociological dimensions, such as anthropology, education, geography, and political science.

On the other side, the subdiscipline has arguably become too reliant on a small number of theories, some of which have been reproduced over three to four generations of scholars with few really significant redevelopments or reconfigurations of the main precepts or arguments. Among the most influential theorists here have been Bourdieu and Foucault, known worldwide in the social sciences; Elias, mainly known and used in the UK and some parts of the European continent; and, Luhmann, best known and understood in Germany and Scandinavia. Notably, with the exception of the even older Elias (1897–1993), these modern theorists were of a largely similar historical period, being born in the interwar period (1920s–1930), and developing their *oeuvres* and *magni opi* in the 1960s through to the 1980s. In other words, their main work was developed some 40–60 years ago, with the apogee in their usage within sociology and the sociology of sport perhaps having been in the late 1980s or early 1990s, some 30 years ago.

We have no doubt that sociologists of sport will continue to draw significantly on these theorists. Indeed, as the space and time allocated to sociology within sport-related degree programmes come under pressure, it becomes more likely that they will be among the few if only social theorists that students encounter to any significant extent. However, we contend that the sociology of sport needs to pursue and to sustain a wider range of theoretical approaches, for the reasons mapped out above, including with respect to the benefits of maintaining a cosmopolitan and glocal array of standpoints, and to enhance the subdiscipline's vitality and capacity to respond to fresh research challenges. Thus, looking forward a further 20–30 years, to the 2040s–2050s, sociologists of sport should aspire to engage with a wider array of theorists and theoretical frameworks, keeping in mind that the primary works of the quartet above would by that point be some 60–90 years old, and in the case of Elias (1939) (38), even over a century in vintage. As noted earlier, lack of theoretical variation and renewal would leave the sociology of sport more open to appearing staid and entropic to those in sociology or wider social science. In turn, it would weaken our appeal in terms of securing research funding, or being invited into multi-disciplinary research collaborations.

### Transnational coordination

Given its challenging circumstances, sociologists of sport across the world need to do all they can to transform and enhance the transnational constitution and coordination of their global field. Three key points follow here.

First, the transnational sociology of sport continues to be dominated by the Anglophone global North, most obviously involving North America, the UK, Australasia, and Anglophone scholarship in Europe, East Asia, and elsewhere. This transnational field has far more to do in order to engage fully with actual, emergent, and potential scholarship across the vast diversity of low- and middle-income countries. Such an engagement is vital if the sociology of sport is to be a genuinely “global” field. It is also vital if the subdiscipline is to observe, through a kind of collective self-practice, its own incessant and ubiquitous demands for all institutions in sport to tackle fundamental issues of marginalization, colonization, and decolonization. This would enable the subdiscipline to rethink its ontological, epistemological, methodological, and substantive dimensions in ways that fully engage LMIC and non-Anglophone perspectives. Moreover, it is essential that we recognize the vast social divisions and inequalities *across* the global South; hence, for example, we must do all we can to ensure that the social scientific “voices” of the “global South” are not purely or primarily those of national or regional elites.

A particular problem of international collaboration, however, lies in what we might term the language and the ontology of publication. For many years, the Anglophone research community took little notice of research in other countries. This is, of course, because representatives from Anglophone countries have had no need to adopt another language for international discourse. However, in so many other countries—for example France, Spain, Germany, and Poland in Europe; Brazil, Argentina, and Chile in South America; China, Japan, South Korea, and Taiwan in East Asia—research projects were and continue to be conducted, books written, and articles published, but in the local languages. The increase in the importance of world rankings for the self-image of universities and the increasingly demanded internationalization of research cooperation has led to a rethinking of academic work (including in the social sciences) in these countries. Now, English is increasingly the lingua franca of scientific communication for these countries as well. And yet, there is a large number of highly interesting research results that have not been published in English and will never find their way into the international sport sociology community if they are not translated. At the same time, academics in many of these countries argue that the Anglophone ontologies of writing or publishing in the social sciences—particularly for journal articles, but also for larger works such as PhD theses—are very different to the approaches found in their home nations. Again, there is a concern that global sociology may become too homogenized, and undermine its glocal diversity, if scholars in Anglophone countries fail to recognize significant cultural differences in how sociology and other social sciences are “done” in non-Anglophone and/or global South contexts.

Second, the principles behind the points above—centred on tackling tendencies towards homogenization and marginalization *within* the subdiscipline—apply across the world, including of course in the global North. Equality, diversity and inclusion (EDI) concerns must be directed onto the subdiscipline in full, and that means by looking beyond “acknowledgements of privilege”, to continue to press higher education institutions to redistribute resources such as studentships, posts, research grants, leadership roles, and academic status. Thus, the sociology of sport is ripe for transformation with regard to repairing the consequences of social divisions along the lines of class, gender, ethnicity, race, disability, and, as flagged above, North/South and Anglophone/Non-Anglophone divisions.

Third, transnational networks and associations need to identify ways in which the subdiscipline can become far more coherent and coordinated, to tackle tendencies towards fragmentation. The most obvious area lies in respect of the international associations for sociologists of sport, such as EASS, ISSA, NASSS, 3SLF, and also the various national or regional associations and networks within the subdiscipline, such as in different parts of Europe, East Asia, and Oceania.^7^ Currently, each association tends to engage particular clusters of academics, with some overlaps. However, we find that North American academics tend not to attend conferences in Europe hosted by EASS/ISSA in the summer, while NASSS conferences (staged in November) tend to attract a relatively limited cohort of European academics, especially non-Anglophone ones. It is vital that these associations, particularly through their leadership groups, explore ways to facilitate more effective communication and coordination. The benefits here would include greater volume of interaction and exchanges between individuals and research groups across these diverse associations and networks; and, a stronger cross-fertilization of research ideas, networks, and projects. This would also enable associations potentially to co-stage events—as we saw with the EASS and ISSA joint conference in Tübingen in 2022—and it would also avoid the particularly counter-productive occasions, which have happened twice in recent years, when two international associations have staged their own conferences at the same time as each other. Further, a focus on international associations and conferences would draw sociologists of sport to reflect on how they may engage with other associations, whether these are more all-encompassing ones (such as the European College of Sport Sciences, which includes a significant social science dimension), or more disciplinary specific ones (such as those in sport management, physical education, sport history, sport philosophy, sport economics, and so on).

### More horizontal and less vertical collaborations

There needs to be a better balance between vertical and horizontal types of networking and collaboration in the sociology of sport. By “vertical”, we mean hierarchical collaborations, mainly between academics at senior (e.g., professor), mid-career (e.g., associate professor), early career (e.g., assistant professors, postdoctoral research associates), and doctoral researcher levels. Conversely, “horizontal” refers to collaborations among academics at the same level, such as between early career researchers or between PhD students.

We recognize that the volume and variety of vertical collaborations have grown substantially over the last two or three decades. Doctoral researchers and their supervisors now co-author many more papers than in the past, in ways that are coming to mimic the formats found with colleagues in the natural sciences. We find that funded research projects often feature teams of researchers, usually led by an established academic, with early career and doctoral researchers also on board with the role of collecting and analysing data. We appreciate there are further structural and cultural reasons for these hierarchies. In some countries, university employment and departmental structures are set up with Chairs (professors) at the centre, supported by collaborating clusters of more junior colleagues. Younger academics may also seek to work with specific senior colleagues, developing their research skills, publication profiles, and, crucially in many contexts, professional networks in ways that enhance future employment and career-building opportunities. On occasion, however, these vertical relations can inhibit the academic development and personal freedoms of younger colleagues, such as when senior staff act almost as conservators with their early career and doctoral researchers, controlling which other academics they can talk to, or restricting their freedom of association at conferences.

In our view, this verticality in academe needs to be balanced by a much greater focus on horizontal collaborations, particularly with doctoral and early career researchers. More horizontal collaborations of this kind would help to enhance the vitality of the sociology of sport; the exploration of new theories, methodologies, and substantive areas of research; and, the array of interdisciplinary and international partnerships across the subdiscipline. These horizontal forms of networking enable young academics to gain valuable experience in genuinely collaborative, creative research projects and publishing; to build new networks and communities of colleagues internationally; and, to share their accounts, experiences, and perspectives with peers at similar stages of career development.

It is worth recalling that, from the late 1960s onwards, it was groups of young academics at similar career stages who undertook much of the foundational work within the sociology of sport, and also who led much of the adventurous development of new research paradigms across the subdiscipline. Such horizontal collaborations among young scholars would help to revitalize the sociology of sport in this way. Of course, to facilitate this process, more powerful, senior staff would at least be required to take a step back, or, better still, to positively encourage and enable such peer-based collaborations.

### Public engagement

Famously, CW Mills (1959) (39) argued that a defining feature of the sociological imagination was the capacity to view “personal troubles” as “public issues”, that impact on many people, and which are shaped by diverse structural factors and cultural processes. From this, we may consider how this sociological imagination may be fostered and harnessed by sociology, and the sociology of sport, in ways that enhance their social relevance and public engagement. Crucially, if sociology is to enhance its public engagement, it has no choice but to break away from a pure observer role and to develop greater competence in the translation of its results. In this context, contact with both politics and sports practice plays an important role.

Public engagement takes many forms, including advising leading decision-makers and other officials within key organizations; working with organizations to enhance their policy and practice; and, contributing to debates in the public sphere (e.g., through mass and social media). The easiest way of doing this latter form of public engagement is through short articles in media open to sociological contributions; the online outlet, *The Conversation*, provides an obvious example. These outputs may accumulate many “reads” or “clicks”, and may enable PhD students and early career researchers to put down markers for their research and academic presence, but the extent to which they have direct non-academic influence or impact is very much open to debate. On the other side, perhaps the most fully impactful approach is to ensure that sociologists are able to take positions on scientific advisory bodies and other such panels, which feed directly into policymaking at national and international levels. Further impactful and direct modes of external engagement include organizational collaborations, which may involve the “co-creation” of research projects, and the translation of findings into fresh strategies, policies, and practices for the outside partner.

There is a long-term trend for national and international research foundations to direct social scientists towards these types of external collaboration or impact in order to secure research funding. Hence, sociologists would do well to build these links in the pursuit of funding. We should recall also that these external partners take many forms. Certainly, sport clubs and governing bodies, governmental bodies (local, national, and international), and corporations are included here, but so too are NGOs, campaign groups, social movements, and other agencies that are perhaps more likely to engage directly with, and to champion the causes of, marginalized social groups, and which perhaps also offer relatively close fits with the theories and perspectives that are held by some sociologists. In many universities—especially for sociologists and other academics holding privileged positions within “research-intensive”, low-teaching institutions—the pursuit of this research funding is a strategic necessity. Failure to do so serves mainly to marginalize further the discipline in terms of securing its requisite share of research funding, its relevance or influence with external organizations and publics, and its future within higher education; otherwise, university leaders will inevitably be required to ask: why invest in this discipline, and not in others that are willing to pursue funded research and external impact?

## Conclusion

Our aim here has been to examine critically the academic and wider societal position of the sociology of sport, and to advance specific ways forward (or “new horizons”) for the subdiscipline. We have argued that social science, sociology, and the sociology of sport hold comparatively peripheral positions—which we have termed *periphery^1,2,3^* respectively—within academe and more broadly; indeed, much of the subdiscipline's marginality derives from its location within these wider academic milieux. In contrast, we also highlighted a range of strengths and advantages that sociology and the sociology of sport possess within academic and wider, non-academic fields. These two sections provided the critical context for our discussion of routes ahead for the sociology of sport, specifically in improving its positioning within academe, scaling up to produce large-scale research collaborations, embracing and building upon its glocal and cosmopolitan aspects, enhancing transnational coordination, advancing horizontal collaborations, and strengthening public engagement.

To conclude, we put forward three main points. First, our intention has been to advance an analysis that is critically realistic and plausibly aspirational with regard to the contemporary position and future possibilities of the sociology of sport, particularly within the academic context. In doing so, we have sought to exercise the type of critical reflexivity that is broadly advocated in much of sociology and the sociology of sport, and to refer this back onto the discipline and subdiscipline themselves. In our view, this type of concerted critical reflection is essential for the future development of any subdiscipline within sport studies, whether these might be located within the social or natural sciences. Hence, we would encourage scholars in diverse fields such as sport biomechanics, geography, history, management, medicine, nutrition, physiology, political science, and psychology also to reflect critically on their respective conditions, positions, and future possibilities, within academe and beyond. Many of the key themes that we highlight here—such as the relative positioning of the subdiscipline within academe, its transnational coordination, and public engagement—may be relevant and applicable to such critical assessments.

Second, our analysis is ultimately directed towards enhancing the sociology of sport, particularly within the academic realm. Sociology has a critical role to play in the full gamut of interdisciplinary research fields within and beyond sport. As we have argued, we do not work in the most auspicious circumstances: disciplines such as psychology and biology tend to have greater prominence, and at times to display a degree of triumphalism, within many research fields. Yet, as the Covid-19 pandemic alone has demonstrated, there is an essential need to look beyond the biological and the psychological, and to examine the sociological dimensions of any research issue.^8^ At the same time, a critical task for sociologists within sport and other fields is to adapt and to reposition the discipline, in the ways that we have outlined, to secure its necessary centrality within the academy and beyond.

Third, in this context, we would also like to emphasize once again that even the most advanced empirical methodology for capturing psychological, biological, and social patterns of human coexistence is no substitute for theory-led, critical sociological reflection. Big data research provides a current example for the irreplaceability of critical sociological reflections where they are increasingly being considered as unnecessary. The number of researchers who are convinced that collecting tons of behavioural or communicational data from millions of people automatically leads to “the truth” is continuously rising. Using big data research techniques to analyse patterns of social interactions, collective behavioural patterns, or consumer trends, certainly means progress for certain types of studies in social science studies, considering the chaos of societal communication. However, this does not mean that critical thinking, and particularly a critical theory-driven sociological analysis, has become useless. On the one hand, pure big data approaches have the disadvantage that “no matter their “depth” and the sophistication of data-driven methods (…) in the end they merely fit curves to existing data” (41). To give one example (42): even if it is possible to collect billions of data about sentiments of football fans' tweets, the findings regarding collective emotionality in football still remain superficial if the tweets cannot be contextualized against the background of discursive strategies on Twitter, emotional contagion in larger groups, the typical “language” of fans in this sport (or in other words, theoretical sociological reflections on the dynamics of collective emotions in sports), as well as the large-scale, social structural processes (such as globalization, commodification, securitization, mediatization, and postmodernization) that have reshaped elite-level global football over the past few decades (43). On the other hand, to avoid an uncritical approach to the results of big data surveys, it is necessary to figure out “the sociotechnical processes involved along the “data building chain”” (44). Data does not just appear out of thin air. They build on previous research, but they are also influenced by existing actor constellations in the relevant research field, by power relations in scientific circles, and, last but not least, by scientific trends. Research, including big data research, is therefore always characterized by a pre-selection of questions, variables and study populations, which in turn depend on the social context in which they are “created”.

Sociological thinking, it can be said, is therefore not replaceable, either in science in general or in sports science in particular. On the contrary: in a world in which it is possible to manipulate publics *via* social networks, in which political pressure can influence the selection of research questions that are publicly considered relevant, and in which complexity is a central characteristic of every world problem, critical sociological thinking is even more important than ever.

## Footnotes

^1^Triplett (2) – likely less familiar to sociologists – wrote what is widely considered to be the first study in sport psychology.

^2^Interview for *Women’s Own* magazine, published 1 October 1987.

^3^In the UK, many sociology departments have established criminology programmes. One of us recommended and planned out a full criminology undergraduate programme in the mid-2000s for a sociology section at a university in Scotland, but this failed to gain the support of the section head. 15 years later, a criminology undergraduate programme was established by that same sociology section.

^4^We have in mind here the communal atmosphere at the joint European Association for Sociology of Sport (EASS) and International Sociology of Sport Association (ISSA) conference at the University of Tübingen in June 2022.

^5^See for example Collison et al. (35).

^6^On glocalization as a theory and social process, see Robertson (37).

^7^These refer to European Association for Sociology of Sport (EASS), International Sociology of Sport Association (ISSA), North American Society for the Sociology of Sport (NASSS), and Société de Sociologie du Sport de Langue Française (3SLF).

^8^See for example the arguments of Connell (40) on the role of sociology with respect to Covid-19.

## References

[1] Bette KH. Sportsoziologie. In: Kneer G Schroer M, editors. Handbuch spezielle soziologien. Wiesbaden: VS Verlag für Sozialwissenschaften (2010) p. 587–604.

[2] Triplett N. The dynamogenic factors in pacemaking and competition. Am J Psychol. (1898) 9:507–33. 10.2307/1412188

[3] Descartes R. Meditations on first philosophy: with selections from the objections and replies. (translated by Michael Moriarty). Oxford: Oxford University Press (2008).

[4] Marzano M. La philosophie du corps. Paris: Presses Universitaires de France (2007).

[5] Elias N Dunning E. Dynamics of sport groups with special reference to football. Br J Sociol. (1966) 17:388–402. 10.2307/589186

[6] Edwards H. The revolt of the black athlete. New York: Free Press (1968).

[7] Edwards H. Sociology of sport. Homewood, IL: Dorsey (1973).

[8] Heinilä K. Notes on the inter-group conflicts in international sport. Int Rev Sport Sociol. (1966) 1:31–40. 10.1177/101269026600100103

[9] Kenyon G Loy J. Toward a sociology of sport: a plea for the study of physical activity as a sociological and social psychological phenomenon. J Health Phys Educ Recreation. (1965) 36:24–5, 68–69. 10.1080/00221473.1965.10616965

[10] Klein M Christiansen G. Gruppenkomposition, Gruppenstruktur und Effektivität von Basketballmannschaften. In: Lüschen G, editors. Kleingruppenforschung und gruppe im sport. Cologne: Westdeutscher Verlag (1966). p. 181–91.

[11] Lüschen G. (ed.) Kleingruppenforschung und gruppe im sport. Cologne: Westdeutscher Verlag (1966). p. 181–91.

[12] Lüschen G. Cooperation, association, and contest. J Conf Resolut. (1970) 14(1):21–34. 10.1177/002200277001400103

[13] McIntosh P. Sport in society. London, England: C.A. Watts (1963).

[14] Rigauer B. Sport und arbeit. Frankfurt: Suhrkamp (1969).

[15] Dunning E. Some conceptual dilemmas in the sociology of sport. In: Albonico R Pfister-Binz K, editors. Sociology of sport: Theoretical foundations and research methods. Basel: Birkhäuser (1972) p. 331–45.

[16] Dunning E. Sport matters: sociological studies of sport, violence, and civilization. London: Routledge (1999).

[17] Thing LF Vilanova A Connolly P Toms M Thiel A. Editorial. Eur J Sport Soc. (2015) 12(3):1–3. 10.1080/16138171.2015.11687965

[18] Caspersen CJ Powell KE Christenson GM. Physical activity, exercise, and physical fitness: definitions and distinctions for health-related research. Public health reports. (1985) 100(2):126-31. 3920711 PMC1424733

[19] Gouldner AW. The coming crisis in western sociology. New York: Basic Books (1970).

[20] Bernard LL. The significance of comte. Soc Forces. (1942) 21(1):8–14. 10.2307/2570423

[21] van den Hoonaard WC. The seduction of ethics. Transforming the social sciences. Toronto: University of Toronto Press (2011).

[22] Israel M Hay I. Research ethics for social scientists: between ethical conduct and regulatory compliance. London Etc.: Sage (2006).

[23] Zussman R. Sociological perspectives on medical ethics and decision-making. Annu Rev Sociol. (1997) 23(1):171–90. 10.1146/annurev.soc.23.1.171

[24] Nature (Editorial). Time for the social sciences. Nature. (2015) 517:5. 10.1038/517005a 25557694

[25] Walport M Beddington J. Innovation: managing risk, not avoiding it. Government Chief Scientific Adviser's annual report for 2014. (2014). Available from: https://www.gov.uk/government/uploads/system/uploads/attachment_data/file/381905/14-1190a-innovation-managing-risk-report.pdf.

[26] Berger P. Einladung zur Soziologie. Eine humanistische Perspektive. Konstanz: UVK (2011).

[27] Eickelpasch R. Grundwissen Soziologie. Ausgangsfragen, Schlüsselthemen, Herausforderungen. Stuttgart: Klett (1999).

[28] Rowe D. Sports journalism: still the “toy department” of the news media? Int Rev Sociol Sport. (2007a) 45(3):355–71. 10.1177/1012690210366792

[29] Weber M. Gesammelte Aufsätze zur Wissenschaftslehre. Tübingen: Mohr (1922).

[30] Thiel A Mayer J. Characteristics of voluntary sports clubs management: a sociological perspective. EurSport Manage Q. (2009) 9(1):81–98. 10.1080/16184740802461744

[31] Thiel A Munz B. Individualized training as a biopsychosocial challenge – interlinking epigenetics, social sciences and psychology in sport scientific research. Eur J Sport Soc. (2018) 15(3):211–5. 10.1080/16138171.2018.1500116

[32] Landecker H Panofsky A. From social structure to gene regulation, and back: a critical Introduction to environment epigenetics for sociology. Annu Rev Sociol. (2013) 39:333–57. 10.1146/annurev-soc-071312-145707

[33] Landecker H. Food as exposure: nutritional epigenetics and the new metabolism. BioSocieties. (2011) 6:167–94. 10.1057/biosoc.2011.1 23227106 PMC3500842

[34] Loi M Del Savio L Stupka E. Social epigenetics and equality of opportunity. Public Health Ethics. (2013) 6(2):142–53. 10.1093/phe/pht019 23864907 PMC3712403

[35] Collison H Darnell S Giulianotti R Howe PD. (eds) Routledge handbook of sport for development and peace. London: Routledge (2018).

[36] Jungert M Romfeld E Sukopp T Voigt U. (eds) Interdisziplinarität. Theorie, praxis, probleme. Darmstadt: Wissenschaftliche Buchgesellschaft (2010) p. 25–44.

[37] Robertson R. Glocalization: time-space and homogeneity-heterogeneity. In: Featherstone M Ritzer G Robertson R, editors. Global modernities. London: Sage (1995).

[38] Elias N. Über den Prozess der Zivilisation. 2 Bde. Basel: Haus zum Falken (1939).

[39] Mills CW. The Sociological Imagination. Oxford University Press, New York (1959).

[40] Connell R. COVID-19/Sociology. J Sociol. (2020) 56(4):745–51. 10.1177/1440783320943262

[41] Coveney P Dougherty E Highfield R. Big data need big theory too. Philos Trans: Math, Phys Eng Sci. (2016) 374:1–11. 10.1098/rsta.2016.0153 PMC5052735 27698035

[42] Spaaij R Thiel A. Big data: critical questions for sport and society. Eur J Sport Soc. (2017) 14(1):1–4. 10.1080/16138171.2017.1288374

[43] Giulianotti R Numerato D. Global sport and consumer culture: an Introduction. J Consum Cult. (2018) 18(2):229–40. 10.1177/1469540517744691

[44] Capogna S. Sociology between big data and research frontiers, a challenge for educational policies and skills. Qual Quant. (2022) 5:1–20. 10.1007/s11135-022-01351-7 PMC8897736 35283540

